# Initiation of HIV Reverse Transcription: Is Enzyme Flipping Required?

**DOI:** 10.3390/v3040331

**Published:** 2011-04-12

**Authors:** Matthias Götte

**Affiliations:** 1 Department of Microbiology and Immunology, McGill University, Duff Medical Building (D-6), 3775 University St., Montreal, QC, H3A 2B4, Canada; E-Mail: matthias.gotte@mcgill.ca; Tel.: +1-514-398-1365; Fax: +1-514-398-7052; 2 Department of Biochemistry, McGill University, Montreal, QC, H3G 1Y6, Canada

**Keywords:** HIV, reverse transcription, initiation

## Abstract

Liu and colleagues have recently studied dynamic changes in the orientation of HIV reverse transcriptase (RT) on its nucleic acid substrate during initiation of DNA synthesis. The authors employed a single molecule FRET assay and revealed the existence of an equilibrium between polymerase-competent and “flipped” polymerase-incompetent orientations. RT flipping correlates with enzyme pausing during initiation, while the transition to the processive elongation phase correlates with increases in the population of polymerase-competent complexes. The potential biological significance of these findings is discussed in this commentary in lieu of the entire process of reverse transcription.

The initiation of human immunodeficiency virus (HIV) reverse transcription is a complex reaction that converts the single-stranded RNA genome into double-stranded DNA. Like other retroviruses, HIV recruits a cellular tRNA to initiate DNA synthesis. HIV utilizes human tRNALys3 that forms a specific complex with the viral RNA and its primer binding site (PBS). 18 residues at the 3′-end of tRNALys3 are complementary to the PBS, which provides a substrate for the virally encoded reverse transcriptase (RT). The RT enzyme is multifunctional and possesses DNA polymerase activities on both DNA and RNA templates, and a ribonuclease H (RNase H) activity that degrades the transcribed RNA template of DNA/RNA replication intermediates. Both active sites are separated by a distance that corresponds to a duplex of 18–19 base pairs in A conformation. Thus, the polymerase initially interacts with an RNA/RNA duplex and gradually moves into newly synthesized DNA/RNA hybrids. Enzyme kinetics revealed that the first nucleotide incorporation events are slow and the corresponding complexes are fragile, while DNA synthesis is more efficient and robust following a transition to elongation as HIV RT accommodates significant regions of DNA/RNA [1]. At the same time, the RT enzyme needs to overcome RNA secondary and/or tertiary structures in this process.

Liu *et al.* add yet another layer of complexity to the reaction [2]. The authors have elegantly demonstrated that HIV RT can adopt two different orientations when bound to the tRNALys3— RNA(PBS) complex: a polymerase-competent configuration and a polymerase-incompetent, or “flipped” configuration. The authors employed a previously validated single molecule fluorescence resonance energy transfer (FRET) assay [3,4] in an attempt to study the dynamics of RNA binding during initiation. A FRET acceptor (Cy5) was introduced at a strategic position of a model viral RNA(PBS), while HIV-1 RT was labeled with a FRET donor (Cy3). In this particular set-up, a high FRET value is indicative for the polymerase-competent complex. Here, the polymerase active site interacts with the 3′ end of the primer, while the RNase H active site is located over the RNA template. A low FRET signal is indicative for the flipped configuration in which the RNase H active site is positioned in close proximity to the 3′ end of the primer and the polymerase active site is located over the template. Although it is difficult to determine the location of RT at single nucleotide resolution with FRET-based technologies, previous RNase H mapping experiments help to characterize the polymerase-competent complex [5]. Weak RNase H mediated cuts can be observed at a distance of 18 to 19 base pairs upstream of the primer terminus, just at the end of the RNA/RNA duplex.

The observation that HIV-1 RT can bind its various nucleic acids substrates in (at least) two different orientations is not novel *per se*; however, it is somewhat unexpected to see a significant population of the flipped configuration at early stages during initiation of the first DNA strand (or minus strand DNA). RNase H mapping experiments, high-resolution footprinting, and also previous FRET studies on substrates that mimic the initiation of the second DNA strand (or plus strand DNA) have already revealed the existence of the two complex populations [4,6,7]. The initiation of plus strand DNA requires an RNA primer, referred to as polypurine tract (PPT), that is later cleaved from newly synthesized DNA. In this case, both the polymerase and RNase H activities are directed against the same strand, and flipping orientations provides a plausible mechanism that facilitates a temporal coordination between DNA synthesis and the primer removal reaction. A pausing site at position +12 appears to trigger the flip in orientations [7]. This temporal stop in DNA synthesis correlates with RNase H cleavage in close proximity of the RNA-DNA junction. Liu and colleagues now report a correlation between specific pausing during initiation of minus-strand DNA synthesis with a flip in orientations of RT binding [2]; pausing at position +3 and +5 correlate with an increase in the flipped orientation. Of note, their single molecule approach provides strong evidence to suggest that enzyme dissociation is not required for the flip and the two distinct orientations characterize the bookends of a dynamic equilibrium.

The authors also devised mutated RNA templates to study the impact of defined secondary structures on enzyme pausing and the release from pausing sites, respectively. Changes in FRET patterns during early steps of the initiation reaction suggest that a template associated stem-loop structure, just ahead of the polymerizing enzyme, triggers the flip, and, in turn, enzyme pausing. Strand displacement during active DNA synthesis can eventually disrupt the stem-loop and the RT enzyme transitions into elongation. The efficient elongation phase beyond this point correlates with a marked increase in the population of polymerase competent complexes. Moreover, the viral nucleocapsid (NC) protein, that is implicated in facilitating the initiation of reverse transcription at various stages [8], appears to destabilize the stem-loop structure. This observation is in agreement with chemical propping experiments [9].

Overall, this model is intriguing as it logically translates structural dynamics into function. However, at the same time, it raises the question whether/how the virus benefits from RT flipping during minus-strand initiation. Flipping is detrimental to efficient DNA synthesis, so why would HIV evolve to slow down the start of the reaction? A coordinated change in orientations is evident and plausible at the level of plus-strand initiation that involves initiation of DNA synthesis from an RNA primer and its subsequent RNase H mediated removal. In contrast, minus-strand initiation is not linked to the removal of the tRNA primer that occurs at a later stage of reverse transcription. The authors speculate that a slow initiation reaction may prevent DNA synthesis before the release of viral particles. Circumstantial evidence suggests that artificial increases in efficiency of reverse transcription may result in reduced infectivity, which appears to provide some support for this notion. However, the exact timeline that involves tRNA-RNA (PBS) duplex formation, processing of precursor proteins Gag/Gag-Pol, the release of the mature RT, and ultimately the formation of a productive ternary complex has yet to be determined.

Although DNA synthesis can be demonstrated in isolated virions, the yield remains low and the low concentrations of available nucleotide pools provide a plausible explanation for this observation [10]. Low concentrations of dNTPs also correlate with increases in RT flipping. Thus, flipping could be a consequence rather than a cause of inefficient nucleotide binding and incorporation at this stage. However, the change in orientations of RT may help to protect the PBS from premature degradation, which would be detrimental to the entire process of reverse transcription. Although the RT-associated RNase H activity is specific to RNA/DNA hybrids, the template of a binary tRNA/RNA(PBS) complex is eventually cleaved provided that the components are incubated for longer periods of time [5]. The absence of RT flipping under conditions that limit DNA synthesis through diminished dNTP pools would therefore increase the efficiency of RNase H cleavage on duplex RNA/RNA.

Finally, the initiation of minus-strand initiation is a highly specific reaction and serves as a logical target in drug discovery and development efforts [11,12]. However, the approved nucleoside and non-nucleoside analogue RT inhibitors—NRTIs and NNRTIs, respectively—can theoretically act at any stage of DNA synthesis. Rate-limiting steps, like the initiation of minus- and plus-strand DNA synthesis, are particularly vulnerable to inhibition. Of note, emerging evidence suggests that NNRTIs act predominantly at the level of plus-strand initiation [4,13,14]. An increase in RT flipping can here potentiate the inhibitory effects on nucleotide incorporation, which provides a proof-of-concept. The initiation of minus-strand synthesis may offer different, yet underexplored opportunities for intervention with small molecules. Future studies will need to show whether FRET-based single molecule assays, as described by Liu and colleagues, can be translated in screening platforms that help to identify novel classes of compounds that specifically interfere with dynamic changes associated with RT function.
